# Post-Operative Remote Monitoring for Same-Day Discharge Elective Orthopedic Surgery: A Pilot Study

**DOI:** 10.3390/s21175754

**Published:** 2021-08-26

**Authors:** Vibav H. Mouli, Christopher X. Carrera, Natalie Schudrowitz, Jean Flanagan Jay, Vivek Shah, Wolfgang Fitz

**Affiliations:** Department of Orthopaedic Surgery, Brigham and Women’s Hospital, Harvard Medical School, Boston, MA 02115, USA; vhmouli@med.umich.edu (V.H.M.); ccarrera@bwh.harvard.edu (C.X.C.); nschudrowitz@wisc.edu (N.S.); jflanaganjay@bwh.harvard.edu (J.F.J.); vshah@bwh.harvard.edu (V.S.)

**Keywords:** total joint arthroplasty, TJA, vital sign monitoring, blood pressure, wearable, mobility, biomedical instrumentation, noninvasive

## Abstract

The purposes of this pilot study are to utilize digital remote monitoring to (a) evaluate the usability and satisfaction of a wireless blood pressure (BP) and heart rate (HR) monitor and (b) determine whether these data can enable safe mobilization at home after same-day discharge (SDD) joint replacement. A population of 23 SDD patients undergoing unicompartmental knee arthroplasty (UKA), total knee arthroplasty (TKA), or total hip arthroplasty (THA) were given a cellular BP/HR monitor, with real-time data capture. Patients took three readings after surgery, observing for specific blood pressure decreases, HR increases, or hypotensive symptoms. If any criteria applied, patients followed a hydration protocol and delayed ambulation. Home coaching was also provided to each patient. Patient experience was surveyed, and responses were assessed using descriptive statistics. Of 18 patients discharged (78%), 17 returned surveys, of which 100% reported successful device operation. The mean “ease of use” rating was 8.9/10; satisfaction with home coaching was 9.7/10; and belief that the protocol improved patient safety was 8.4/10. A total of 27.8% (*n* = 5) had hypotensive readings and appropriately delayed ambulation. Our pilot findings support the feasibility of and confirm the satisfaction with remote monitoring after SDD arthroplasty. All patients with symptoms of hypotension were successfully remotely managed using a standardized hydration protocol prior to safe mobilization.

## 1. Introduction

The predominance of growth in volume of total joint arthroplasty (TJA) procedures over the next five years is expected in the outpatient setting [1]. While traditional, inpatient TJA has variable length of stay (LOS), with, at minimum, an overnight hospital stay [2], same-day discharge (SDD), or outpatient TJA, enables patients to be discharged on the very day of surgery after stabilization criteria have been met [3,4,5]. SDD has emerged as a promising model to optimize both convenience and cost, eliciting potential savings of 30%, or up to USD 8500 per patient, driven by decreased surgical floor care, patient meals, inpatient pharmacy, and inpatient physical therapy [6]. Clinically, complications, readmission rates, and patient-reported outcomes are approximately the same in outpatient vs. inpatient TJA when appropriate patient eligibility criteria are followed [7,8,9,10,11,12]. That is, additional inpatient care does not necessarily lead to improved outcomes; at-home recovery with appropriate pain management, outpatient physical therapy, and escalation pathways (as needed) are just as efficacious in certain patients.

Concurrent to this outpatient shift is a growing interest in web-based platforms for follow-up assessments, patient education, seamless communication, and coaching. Devices to collect real-time biometric data have been introduced into clinical care, measuring blood pressure, oxygen saturation, and blood glucose [13,14]. Wearable trackers can monitor post-operative daily step count [15], while physician–patient messaging platforms can monitor wound appearance [16]. In SDD arthroplasty, there is significant opportunity to utilize these digital health tools to optimize post-operative recovery for patients at home. For example, hypotension, or “orthostatic intolerance”, after arthroplasty remains a key impediment to mobilization, posing increased risk of falls, prosthesis fractures, and fainting [17]. Digital health tools that could identify hypotensive patients have yet to be introduced to minimize these risks. One of the key aspects of implementing these tools is usability [18]. Before launching initiatives that incorporate digital health technology, appropriate diligence must be taken to understand the patient experience, predict barriers to adoption, and validate efficacy.

The purposes of this pilot study are to (a) evaluate the usability and satisfaction of a wireless blood pressure (BP) and heart rate (HR) monitor in same-day discharge arthroplasty patients and (b) determine whether these data can add value in the immediate post-operative phase, specifically to enable safe mobilization at home. We hypothesized that patients would be able to effectively and easily use the BP/HR monitor and that a subset of patients would follow a standardized hydration protocol to appropriately delay ambulation and minimize resulting complications such as fall risk at home.

## 2. Materials and Methods

Approval for this study was given by the institutional review board at Brigham and Women’s Hospital (June 2019 #2019P000687), and all participants gave informed written consent.

**Enrollment:** This was a single institution, single-arm experimental pilot study including 23 patients undergoing primary unicompartmental knee arthroplasty (UKA), unilateral total knee arthroplasty (TKA), or unilateral total hip arthroplasty (THA) with anterior approach from 10/2019 to 3/2020 with a single surgeon. Patients were eligible if they were at least 18 years of age and scheduled for same-day discharge (SDD) after their procedure. Determination of eligibility for SDD was based on patient interest, clinical input and assessment from the surgical team, nursing, physical and occupational therapists and clearance from the case manager to ensure appropriate individual support at home. The exclusion criteria for SDD included any of the following medical conditions: diagnosis of COPD; chronic kidney disease with eGFR < 60; uncontrolled Type 2 diabetes or insulin-dependent diabetes mellitus; diagnosis of CHF or CAD and ratio of triglycerides/HDL > 4; more than two medications used for hypertension; history of stroke, deep vein thrombosis (DVT), pulmonary embolism (PE) or use of chronic anticoagulation; patient not opioid-naïve; movement disorder or inability to stand on one leg as determined by physical therapy; obstructive sleep apnea with or without continuous positive airway pressure therapy (CPAP); excessive anxiety with regard to going home. Additional criteria for exclusion included: (a) inability to operate blood pressure monitor, (b) an arm circumference that exceeds the size of the arm cuff or (c) the use of an implanted cardiac device, such as a pacemaker or defibrillator. All participants gave informed written consent.

**Figure 1:**
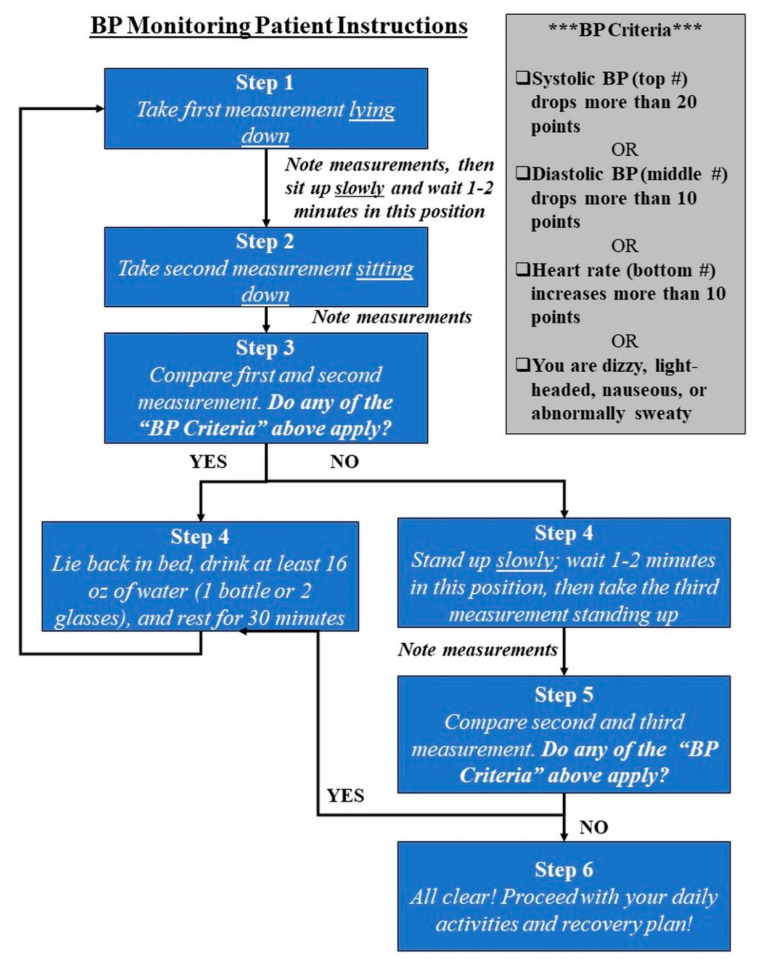
Flow diagram of instructions provided to patients and family.

**Measurements:** Patients were provided with a BodyTrace BP/HR monitor in the Post-Anesthesia Care Unit (PACU), linked to a web-based clinical portal (IGetBetter, Inc., Framingham, MA, USA) for real-time data capture. A member of the research team provided detailed written and verbal instructions during the enrollment process and again in PACU prior to discharge, including a flow diagram guiding the patient through each measurement (Figure 1).

Patients were asked to take three readings (supine, seated, and standing) in the morning after surgery at home, observing for any of the following features indicative of the institution’s protocol for assessing post-op orthostatic hypotension: (a) 20-point systolic drop, (b) 10-point diastolic drop, or (c) 10-point HR increase between readings. Patients were also asked to observe and record any hypotensive symptoms including nausea, dizziness, lightheadedness, and diaphoresis. If any quantitative or symptom criteria applied, patients were instructed to stay in bed, hydrate with a minimum of 16 oz water, rest for 30 min, and repeat the protocol until resolution. Patient readings were automatically uploaded to the clinical portal within approximately 10 s of each measurement. In addition, a patient navigator, a member of our orthopedic physical therapy team, called the patient the morning after surgery to provide one-on-one home coaching. During the call, the navigator validated the BP and HR measurements from the clinical portal with patients’ own noted measurements. He or she also evaluated hypotensive symptoms, assessed hydration levels and mobilization issues, and answered any patient questions. The overall recovery plan was reviewed, and if there was any need for escalation due to insufficient pain management, significant nausea and vomiting, or other abnormal symptoms, the patient navigator would encourage the patient to come into the hospital for evaluation.

**Valuating Patient Experience:** Patients returned to the clinic within 10 days of operation for their first post-op appointment, at which point, they returned their monitor and were asked to rate experience with the monitor and coaching protocol. There were nine questions on the patient experience survey, inclusive of three questions with visual analogue scale (VAS) response options. All responses were assessed using descriptive statistics.

**Outcomes:** The primary outcomes for the study were the ability to follow clinical protocol successfully as measured by “successful operation of the device” (in the patient questionnaires) and patient satisfaction with home coaching (as measured by VAS scores). Secondary outcomes were patients’ beliefs that the protocol would improve safety, the number of patients reporting hypotensive symptoms, and the number of patients who delayed ambulation due to hypotensive symptoms.

## 3. Results

The mean age of all 23 patients enrolled was 65.83 years ± 11.36, with 30.43% female. Of these 23 patients, 18 patients were discharged as planned (78.3% of sample). A summary of descriptive characteristics is presented in Table 1. Five patients who were scheduled and eligible for SDD were admitted overnight due to clinical team decisions by nursing or physical therapy. These patients failed one or more of the following criteria, which are standard of care for hospital discharge: post-operative voiding, climbing stairs, appropriate pain control, safe ambulation with device (cane, crutches, or walker), sensation in extremities after spinal anesthesia, and hemodynamic stability.

**Figure 2:**
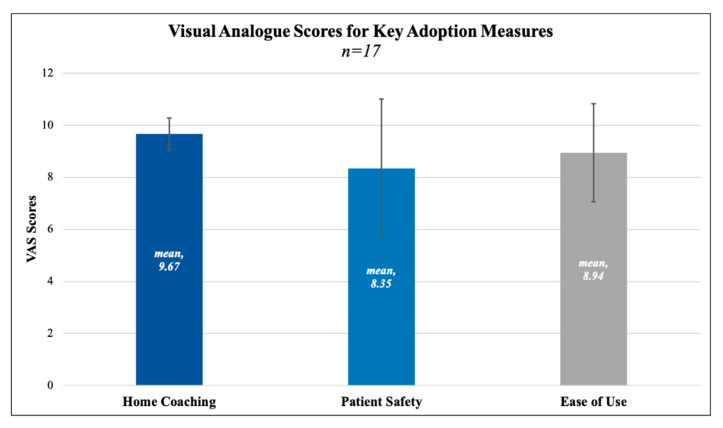
Visual Analogue Scores for Home Coaching, Patient Safety, and Ease of Use. For Home Coaching, 10 represented “perfectly satisfied”; for Patient Safety, 10 represented “extremely likely” that the protocol would improve patient safety; for Ease of Use, 10 represented “perfectly satisfied”.

In the discharged study sample (*n* = 18), mean age was 64.62 years ± 12.09, with 33.3% being female. Seventeen of these patients returned satisfaction surveys (94.44%). All of these patients (*n* = 17) reported successful operation of the device on their survey, secondarily validated by at least three separate measurements on the clinical portal. One patient was discharged but forgot to take measurements and/or follow the protocol. The mean satisfaction rating for “ease of use” was 8.94 out of 10, where 10 represented “perfectly satisfied”. The mean satisfaction rating for home coaching was 9.67 out of 10, where 10 represented “perfectly satisfied”. Mean rating for belief in the protocol improving patient safety was 8.35 out of 10, where 10 represented “extremely likely”. VAS scores and standard deviations are depicted in Figure 2. Six patients provided optional comments on their experience that may inform future optimization efforts, but did not change primary outcomes. There was one patient for whom the data did not transfer to the clinical portal, although measurements were taken and noted physically. This patient did not experience any hypotensive symptoms or meet the criteria for the hydration protocol, as reviewed by our patient navigator. All other data were transferred seamlessly. Results for all survey questions are reported in Table 2.

In the morning after surgery, five patients measured low blood pressure values, higher heart rates, and/or showed hypotensive symptoms (dizziness, nausea, flushed/warmth) and were advised not to get out of bed, to delay mobilization, and to start a standardized hydration protocol. These patients consumed a mean of 14.4 oz water and remained supine in bed for 30 min. Then, they followed a second set of readings. All patients showed resolution of their hypotensive symptoms, and second readings were within normal ranges. All five patients were successfully mobilized without complications such as falls or physical adverse events. No other patient in the study experienced mobilization issues, as reviewed by the patient navigator during the follow-up call.

**Table 2:** Survey provided to patients at 10-day post-operative appointment, including average or breakdown of responses.

Survey Question		Response Options	Average Response
**1**	Were you **able to operate** the BP monitor and cuff successfully?	Yes/No	Yes (100%, *n* = 17) No (0%, *n* = 0)
**2**	How satisfied were you with the **function and ease of use** of the BP monitor and cuff?	Scale of 0–10 (10 being “perfectly satisfied”)	8.94 (SD = 1.89)
**3**	How satisfied were you with the **home coaching and check in call** you received the morning after surgery?	Scale of 0–10 (10 being “perfectly satisfied”)	9.67 (SD = 0.62)
**4**	Did you experience **any of the following symptoms the morning after surgery**? (Please circle all that apply)	Light-Headed Nausea Flushed/Warm None	Yes (17.64%, *n* = 3) No (82.36%, *n* = 14)
**5**	Did you need to take any **additional hydration** in bed before getting up and starting your daily activities? If yes, how much? (e.g., 2 glasses of water, 1 standard bottle of water)	Yes/No Fill-in-the-Blank	Yes (29.41%, *n* = 5) No (70.59%, *n* = 12) Mean = 14.4 Oz additional hydration
**6**	Do you think the BP monitor and cuff will **improve patient safety**?	Scale of 0–10 (10 being “extremely likely”)	8.35 (SD = 2.7)
**7**	Do you think the BP monitor and cuff **should be used on all patients** who are discharged home?	Yes/No	Yes (82.36%, *n* = 14) No (17.64%, *n* = 3)
**8**	Do you think the **home coaching and check in call** by physical therapy should be done on all patients who are discharged home?	Yes/No	Yes (94.12%, *n* = 16) No (5.88%, *n* = 1)
**9**	Do you think **the surgeon** needs to be the one calling the morning after surgery for all patients who are discharged home?	Yes/No	Yes (17.64%, *n* = 3) No (82.36%, *n* = 14)
**10**	Any **additional comments** on your experience? (Optional)	Fill-in-the-Blank	*n* = 6; (full responses not included)

## 4. Discussion

We wanted to see if a cellular BP/HR monitor would add clinical value after same-day discharge arthroplasty. To our knowledge, this pilot is the first study to use remote monitoring technology to address hypotension in outpatient arthroplasty patients, one of the key, modifiable risk factors in post-operative care. In our single surgeon, single-institution study, our findings indicate that patients are able to operate a BP/HR monitor successfully after same-day discharge knee or hip arthroplasty and are very satisfied with both the device and with home coaching. Over 82% of our surveyed sample agree that the BP monitor and cuff should be used on all patients who are discharged home. Several factors likely contribute to this overall positive patient experience. For one, the ability to recover at home, in a familiar setting and with greater individual autonomy is a considerable benefit in this type of RPM-enabled protocol. The simplicity of the device chosen for our study is also noteworthy; the single button on the BodyTrace device was received well in the post-operative phase, as evidenced by high “ease of use” scores. Additionally, this study took advantage of general familiarity with blood pressure and heart rate as core health measurements to facilitate easier adoption. The combination of these features bodes well for future efforts to scale the protocol.

The most important finding in this study was the ability of our protocol to delay ambulation in five out of eighteen patients discharged (27.8%) who demonstrated orthostatic hypotension and/or related symptoms. These elements remain key impediments to mobilization in the acute post-operative period and are more pressing challenges in rapid discharge arthroplasty. In fast-track (i.e., same-day) THA, there is 42% incidence of orthostatic intolerance at 6 h post-op and 19% at 24 h post-op [19]. The number of hypotensive events has been directly correlated with increased LOS in both TKA and THA [20,21], as well as other surgical fields [22,23]. While both aggressive perioperative volume repletion and the increased use of tranexamic acid to limit blood loss are now standard practice [24,25,26], hypotension remains a common cause of rapid discharge failure and complications such as fainting and falls resulting in peri-prosthetic fractures [17]. The only other pilot study in this space, to our knowledge, found improved BP management and mobilization using midodrine hydrochloride, an orally administered vasoconstrictor [27]. In our study, however, we capitalize on non-pharmacological management, utilizing an inexpensive, straightforward means of decreasing risk of adverse consequences via an outpatient hydration protocol.

While this study did not specifically assess cost savings on a line item basis, the protocol holds promise in decreasing financial costs associated with inpatient, post-operative care. We chose a cellular-enabled BP/HR monitor for this study that is relatively inexpensive (approximately USD 100 on the public market); however, there are numerous devices, both cellular and WiFi-enabled, that are less expensive and should be considered in future studies. The utilization of this protocol may support higher quality post-operative care, thereby incentivizing health systems towards a greater number of SDD cases for eligible patients, especially in a post-COVID-19 world.

These findings should be interpreted in light of the study’s limitations. The primary limitation was that the COVID-19 pandemic prohibited the recruitment of further patients, rendering this a pilot. Although our findings are clinically significant for the SDD population, further studies with an increased sample size are necessary before large-scale implementation. Second, as this study was focused primarily on usability and satisfaction, the “real-time” availability of data was only utilized to check compliance and validate noted measurements, not as a means of clinical monitoring. In a future iteration of this study, severe hypotensive readings or symptoms could trigger an automatic notification to the care provider team to prevent adverse events. This requires careful integration into the clinical workflow, as well as clear escalation pathways (e.g., advising patient when to come to the hospital), but may provide added benefit in certain settings.

## 5. Conclusions

Our pilot findings suggest that there is high feasibility, compliance, and patient satisfaction with remote monitoring after same-day total joint arthroplasty. Nearly 30% of our patients demonstrated post-operative hypotension, as monitored with a cellular device. After delayed mobilization and standardized hydration, these patients were successfully remotely mobilized without complications. After further validation, this protocol may start to bridge the existing gap between convenience and quality in same-day discharge recovery.

## Figures and Tables

**Table 1:** The summary of patient characteristics by discharge status.

	Total	Discharged *	Non-Discharged **
**Population** (*n*)	23	18	5
Average Age years (*SD*)	65.83 (11.36)	83.64 (12.09)	70.15 (7.64)
Height cm (*SD*)	175.19 (11.92)	174.18 (11.86)	178.82 (12.75)
Weight kg (*SD*)	87.27 (16.56)	87.27 (17.62)	87.26 (13.72)
BMI kg/m^2^ (*SD*)	28.31 (3.63)	28.61 (3.87)	27.22 (2.59)
**Sex**	*n* (%)		
Male	16 (69.57%)	12 (66.67%)	4 (80.00%)
Female	7 (30.43%)	6 (33.33%)	1 (20.00%)
**Race**	*n* (%)		
White	20 (87.0%)	15 (83.33%)	5 (100%)
Black	2 (8.7%)	2 (11.11%)	0 (0%)
Declined	1 (4.3%)	1 (5.56%)	0 (0%)
**Surgery Type**	*n* (%)		
UKA	10 (43.5%)	10 (55.55%)	0 (0%)
TKA	1 (4.3%)	1 (5.56%)	0 (0%)
THA	12 (52.17%)	7 (38.89%)	5 (100%)

* Patients who underwent SDD and were eligible to participate in the study, ** Patients who were admitted for hospital stay and therefore no longer eligible for study.

## Data Availability

Not applicable.
